# The influence of sexual activity on athletic performance: a systematic review and meta-analyses

**DOI:** 10.1038/s41598-022-19882-2

**Published:** 2022-09-16

**Authors:** Gerald S. Zavorsky, Rebecca A. Brooks

**Affiliations:** 1 Department of Physiology and Membrane Biology, University of California-Davis, Davis, CA USA; 2 Division of Gynecologic Oncology, University of California-Davis, Medical Center, Sacramento, CA USA

**Keywords:** Physiology, Psychology

## Abstract

Several anecdotal reports suggest that sex before competition can affect performance. Our objective was to perform a systematic review and meta-analysis to determine whether athletic performance or some physical fitness measure is affected by prior sexual activity. Web of Science (all databases) and Google Scholar were used to identify studies from which adult healthy subjects were included. As all studies were crossover trials, an inverse variance statistical method with random effects was used to minimize the uncertainty of the pooled effect estimate. Bias was assessed via the revised Cochrane Risk of Bias tool (RoB 2) with a "per protocol" analysis. Nine crossover studies (133 subjects, 99% male) were used in this meta-analysis. All those studies did not examine athletic performance per se, but all studies assessed one or more physical fitness parameters. The RoB 2 suggested that overall, there were some concerns with bias. As there was moderate heterogeneity amongst the different outcomes (Tau^2^ = 0.02, Chi-square = 17.2, df = 8, *p* = 0.03, I^2^ = 54%), a random-effects model was used. The results neither favored abstinence nor sexual activity before a physical fitness test [standardized mean difference = 0.03 (− 0.10 to 0.16), Z = 0.47, *p* = 0.64, where a negative standardized mean difference favors abstinence, and a positive standardized mean difference favors sexual activity]. The results demonstrate that sexual activity within 30 min to 24 h before exercise does not appear to affect aerobic fitness, musculoskeletal endurance, or strength/power.

## Introduction

The long-standing debate surrounding the impact of sexual activity on sports performance has been controversial in mainstream news media over the past several decades^1–9^. This salacious topic has generated widespread discussion, though related high-quality data has been limited. Previous research methodology to assess either positive or negative effects of sexual activity before an event on sexual performance has primarily included subjective assessments from surveys^10–14^. However, those surveys can be biased as these assessments are based on individual beliefs, perceptions, and memory. There have been discussions in book chapters^15^, conference proceedings^16^, and other discussion pieces in journals^17–19^, but objective data, there too, has been lacking.

Several scientific reviews have been published attempting to address this topic^20–24^. Still, some of these reviews have been published in lower impact journals or journals that have been poorly peer-reviewed or not peer-reviewed at all^20–22^, and other reviews do not use metanalytic techniques to synthesize the available data^23,24^. Given the lack of data surrounding this controversial topic, pooling all data from the literature so a proper meta-analysis can occur would be beneficial.

Therefore, the purpose of this paper was to synthesize and evaluate all studies that have examined the influence of sexual activity on athletic performance—or at least the effects of prior sexual activity on some objective measure of physical fitness. Physical performance can be, for example, an objective assessment of aerobic capacity (i.e., a graded exercise test on a treadmill to volitional exhaustion), musculoskeletal endurance (i.e., number of pushups to failure), or a measure of anaerobic power or strength (i.e., one rep max bench press, or peak power assessed from a Wingate test). For this paper, sexual activity was defined as either sexual intercourse or masturbation. Thus, this meta-analysis would fill a much-needed gap in the scientific literature.

## Methods

### Search strategy

The systematic literature review search occurred on February 21st, 2022. GSZ and RAB independently performed the same search strategy, and then the results were examined together. Any disagreement in article selection was critically appraised by GSZ and RAB. A figure that identified the flow of the different phases of the systematic review as recommended by the 2009 PRISMA statement^25^.

Clarivate's Web of Science was one platform used to access multidisciplinary and regional citation indexes; specialist subject indexes; a patent family index, and an index to scientific data sets. Nearly 35,000 journals and books are covered, including proceedings, patents, and datasets. The complete platform includes one hundred eighty-two million records (journals, books, and proceedings). The citation databases in the platform are the following: (1) Web of Science Core Collection (i.e., Emerging Sources Citation Index, Science Citation Index Expanded, Social Sciences Citation Index, Arts and Humanities Citation Index, Conference Proceedings Citation Index, Book Citation Index) (2) BIOSIS Citation Index, (3) BIOSIS Previews, (4) Current Contents Connect, (5) Data Citation Index, (6) Derwent Innovations Index, (7) Korean Journal Database, (8) MEDLINE, (9) Russian Science Citation Index, (10) SciELO Citation Index, and (11) The Zoological Record. "All databases" were selected, and the query was as follows:

- (((((TS = (sexual activity)) OR TS = (sexual intercourse)) OR TS = (masturbation)) OR TS = (coitus)) OR TS = (sexual relations)) AND TS = (athletic performance). From this query, 119 results were found.

Then Google Scholar was used to obtain additional supplementary references. Google Scholar is a freely accessible search engine, but it is not as reliable as Web of Science^26^. The following query was used:

- ("sexual activity" OR "sexual intercourse" OR "masturbation" or "coitus" OR "sexual relations" AND "athletic performance").

The inclusion criteria for this meta-analysis were as follows: (1) studies that used healthy or non-diseased human participants only, (2) published and unpublished studies in any language (including masters and doctoral theses in any language), (3) any conference proceedings or abstracts from conferences in any language, (4) studies that defined sexual activity as intercourse or masturbation, leading to orgasm or not (5) studies that used either a two (or more) group parallel design, or crossover design where subjects served as their own controls, (6) studies that used any type of physical fitness test to assess physical performance, or, studies that measured actual performance in an athletic or sporting event, and finally, (6) studies that presented enough data to obtain a point estimate (e.g., standard error, confidence interval) from each assessment.

Exclusion criteria: (1) non human studies that used animals in the assessment of physical performance, (2) studies that included unhealthy human subjects or subjects with disease, (3) studies that did not display enough data to obtain a point estimate, even after attempting to contact the authors.

### Randomized crossover designs

Studies that examined the influence of prior sexual activity on performance outcomes are likely to use a crossover approach. A crossover approach eliminates variation between individuals and allows each subject to serve as their own control. When an individual receives the experimental and control treatment in a random order (i.e., the randomized sequence), it is labeled a randomized crossover trial. Because both treatments are evaluated for the same individual, the treatment effect can be estimated based on an average of within-individual differences since subjects also serve as their own control. Thus, a crossover trial can theoretically achieve the same precision as a parallel-group trial with only half the sample size, which is further reduced as the variance is smaller within than between individuals^27,28^.

To include a study in a meta-analysis, one would need a point estimate (e.g., relative risk, mean difference) and associated precision of the point estimate (e.g., standard error, confidence interval) from each assessment. Formulas used to calculate the standardized mean difference and standard error of the standardized mean difference in this meta-analysis were from the Cochrane Handbook for Systematic Reviews^29^. Details of these formulas are found in Table S1 in the online supplement.

Physical performance tests were grouped into three categories regardless of there the tests were from the same study or not: (1) aerobic capacity or aerobic fitness (i.e., VO_2max_); (2) musculoskeletal endurance (i.e., number of pushups to fatigue); (3) and muscular power or strength (i.e., handgrip strength, vertical jump height).

A generic inverse-variance approach was used in this study to minimize the uncertainty of the pooled effect estimate^29,30^. The inverse variance method is so named because the weight given to each study is chosen to be the inverse of the variance of the effect estimate (i.e., one over the square of its standard error)^30^. Consequently, larger studies, which have smaller standard errors, are given more weight than smaller studies, which have larger standard errors^30^. Review Manager was used to generate forest plots using the inverse-variance approach (Review Manager (RevMan) [Computer program]. Version 5.4.1). A random-effects model was used as it is anticipated that there is a distribution of true effects since the true effect size might differ from study to study^30,31^. For example, when assessing the effect of sexual activity on a performance outcome, the true effect may be different if the outcome is aerobic capacity versus an assessment of muscular strength/power. Thus, the weight of each physical performance assessment was based on the calculated standard error of the standardized mean differences. That is, studies or assessments with the lowest standard error of the standardized mean differences were weighted more than studies with a larger standard error of the standardized mean differences. In studies where there were multiple outcomes per physical performance category, the average standardized mean difference (SMD) and average standard error of the SMD per study was used.

Inconsistency (*I*^2^) was used to describe the percentage of variability in effect estimates that is due to heterogeneity rather than sampling error^32^. Quantifying inconsistency is defined as

$$I^{2} = \left[ {({\text{Q}}{-}{\text{d}}_{{\text{f}}} )/{\text{Q}}} \right] \times 100,$$

where Q is the chi-square statistic, and d_f_ is its degrees of freedom^30^.

To categorize inconsistency, the following thresholds were used: 0–40% variability was classified as not important; 30–60% variability was defined as moderate heterogeneity; 50–90% was defined as substantial heterogeneity; 75–100% was defined as considerable heterogeneity^30^. A measure of the extent of variation or heterogeneity among the effects of the intervention was calculated as Tau-squared (Tau^2^)^30^. Tau-squared represents the absolute value of the true variance (heterogeneity).

Critical appraisal of each study was determined by a revised version of the Cochrane risk of bias tool (RoB 2)^33^ and its supplement^34^. An excel tool to implement the RoB 2 generated summary assessments of bias^34^. The approach used to assess bias was based on adhering to the intervention described in the trial protocol (the 'per protocol' effect). The analyses were restricted to individuals who adhered to their assigned interventions. GSZ and RAB conducted the bias assessment independently, and then a discrepancy check was implemented to compare assessments. A consensus was then made, and the final bias was reported.

Funnel plots were used to assess publication bias^35,36^ for each physical performance category with adjusted standard errors^37^. Publication bias was also adjusted for missing studies by the Trim and Fill method^38^. Any publication bias would result in an asymmetry of a funnel plot. If publication bias was present, then the smaller studies would show larger effects.

Galbraith plots were made to graphically present the collection of means using z-scores standardized for each physical performance category plotted against its precision (i.e. the reciprocal standard error of z-scores)^39^. This method allows for easy comparison between studies as the units are standardized.

## Results

### Identification of literature-based studies

From the queries generated, 119 results were obtained from the Web of Science platform, and 242 were obtained from Google Scholar, providing 361 references. Five theses were obtained through the literature search^10,40–43^. Two of these theses^40,42^ were not evaluated as they were eventually published in peer-reviewed journals^44,45^. Another thesis was not included in this meta-analysis as it was a survey^10^, and so two theses were used as they had pertinent data^41,43^. One study was orally presented at a congress in 1989^46^, but the data was subsequently published in a journal 11 years later^47^. Thus, only the journal article was included.

The first study that examined the influence of prior sexual activity on physical performance was published in 1968, but the data was not presented in article^48^. We contacted the sole surviving individual that was involved in that study (J.C. Yurick) but he was unable to find the raw data as the study was conducted over 50 years ago^48^. Thus, nine crossover studies totaling 132 male subjects and one female subject were used in this meta-analysis^16,41,43–45,47,49–51^ (Table S2). All studies measured physical fitness outcomes in healthy, physically active men. Furthermore, all studies used a crossover approach. The PRISMA flow diagram illustrates the literature search flow (Figure S1).

Several studies assessed various measures of physical performance in a single session. That is, aerobic fitness, musculoskeletal endurance, and/or strength/power tests were evaluated on the same day. These tests and results are displayed in Table S3. Several calculations were imputed based on the formulas from Table S1.

There were five studies in the aerobic capacity/endurance category. The SD of the mean differences between abstinence and sexual activity conditions were imputed for Sztajzel et al*.*^47^, Boone and Gilmore^49^, and Navarro^43^. Correlation coefficients between both sexual activity and abstinence conditions for those two studies were estimated as the average of two other studies^44,45^ (Table S3).

There were four studies, also, in the musculoskeletal endurance category. The SD of the mean differences between abstinence and sexual activity conditions were imputed for Vajda^41^ and Valenti et al*.*^52^. The correlation coefficients between both sexual activity and abstinence conditions for these two studies, including Vajda and Reguli^51^, were estimated as the value provided by Zavorsky et al*.*^45^ (Table S3).

There were ten assessments from seven studies in the strength/power category. The SD of the mean differences between abstinence and sexual activity conditions were imputed for Kirecci et al*.*^50^, Vajda^41^, Valenti et al*.*^52^, and Navarro^43^, based on the average correlation coefficient of 0.78 between sexual activity and abstinence conditions. The 0.78 correlation coefficient, including Vajda and Reguli^51^, was estimated as the average four assessments from two studies^44,45^ (Table S3).

### Comparison of identified studies

**Figure 1:**
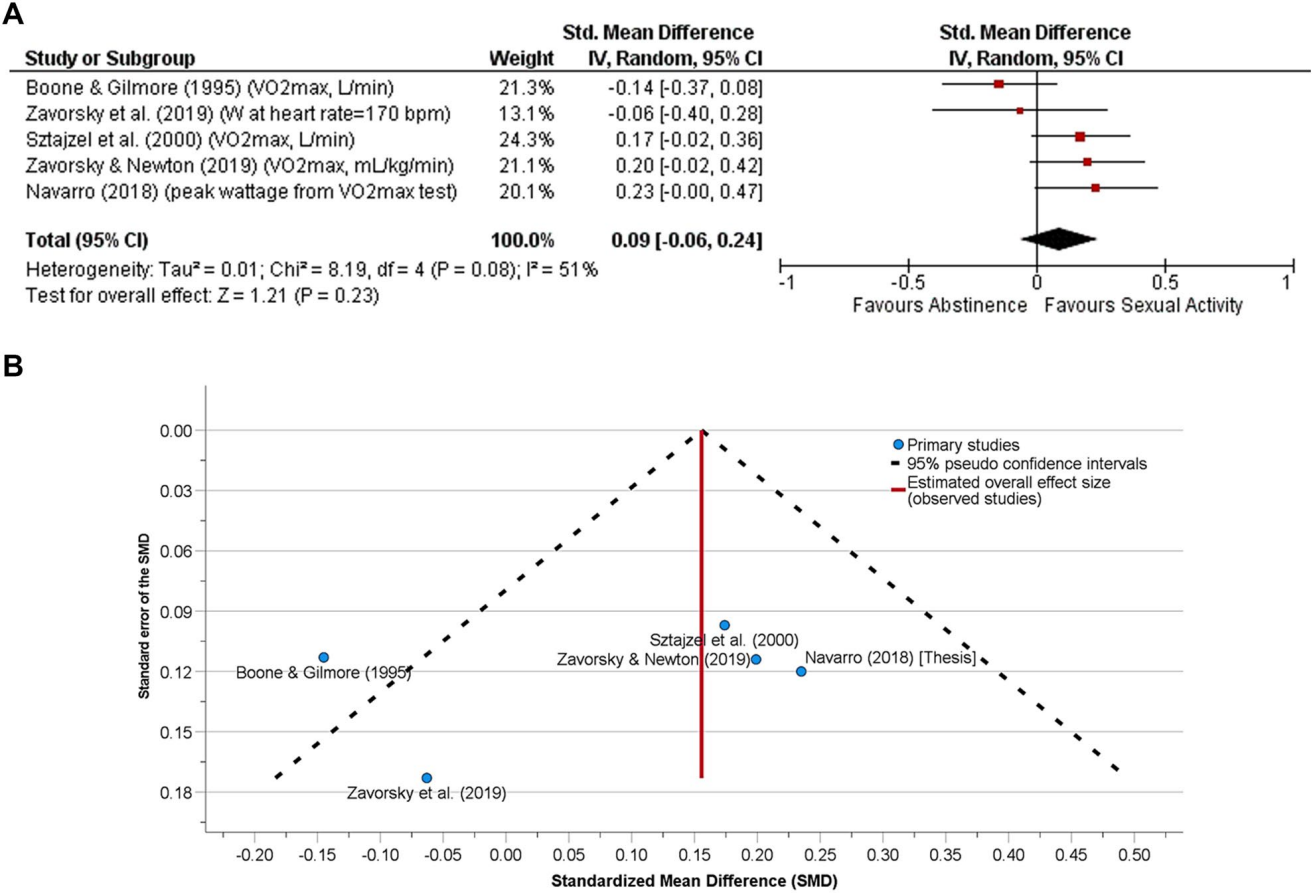
**(A)** The effect of sexual activity that occurred 30 min to 12 h before an aerobic fitness assessment is displayed using a Forest Plot. The data demonstrate that aerobic capacity (aerobic fitness) is unlikely affected by prior sexual activity or abstinence. That is, both experimental and control conditions provide similar test results. A random-effects model was used. No other adjustments were made here. There was moderate heterogeneity between studies (*p* = 0.08). **(B)** Funnel plot for the detection of publication bias in (**A**). Egger’s random effects meta-regression-based test was used with the Knapp-Hartung SE adjustment. There was no funnel plot asymmetry (Intercept = 0.40, SE = 0.43, t = 0.93, *p* = 0.42, 95% CI of the coefficient = − 0.97 to 1.76); Standard error of the effect size = − 2.57, SE = 3.54, t = − 0.73, *p* = 0.52, 95% CI − 13.84 to 8.69). After adjusting for the missing studies, the SMD (SE of the SMD) was 0.16 (0.09) [95% CI − 0.08 to 0.39, *p* = 0.15], demonstrating that neither abstinence nor sexual activity prior to exercise affects aerobic fitness assessment.

**Figure 2:**
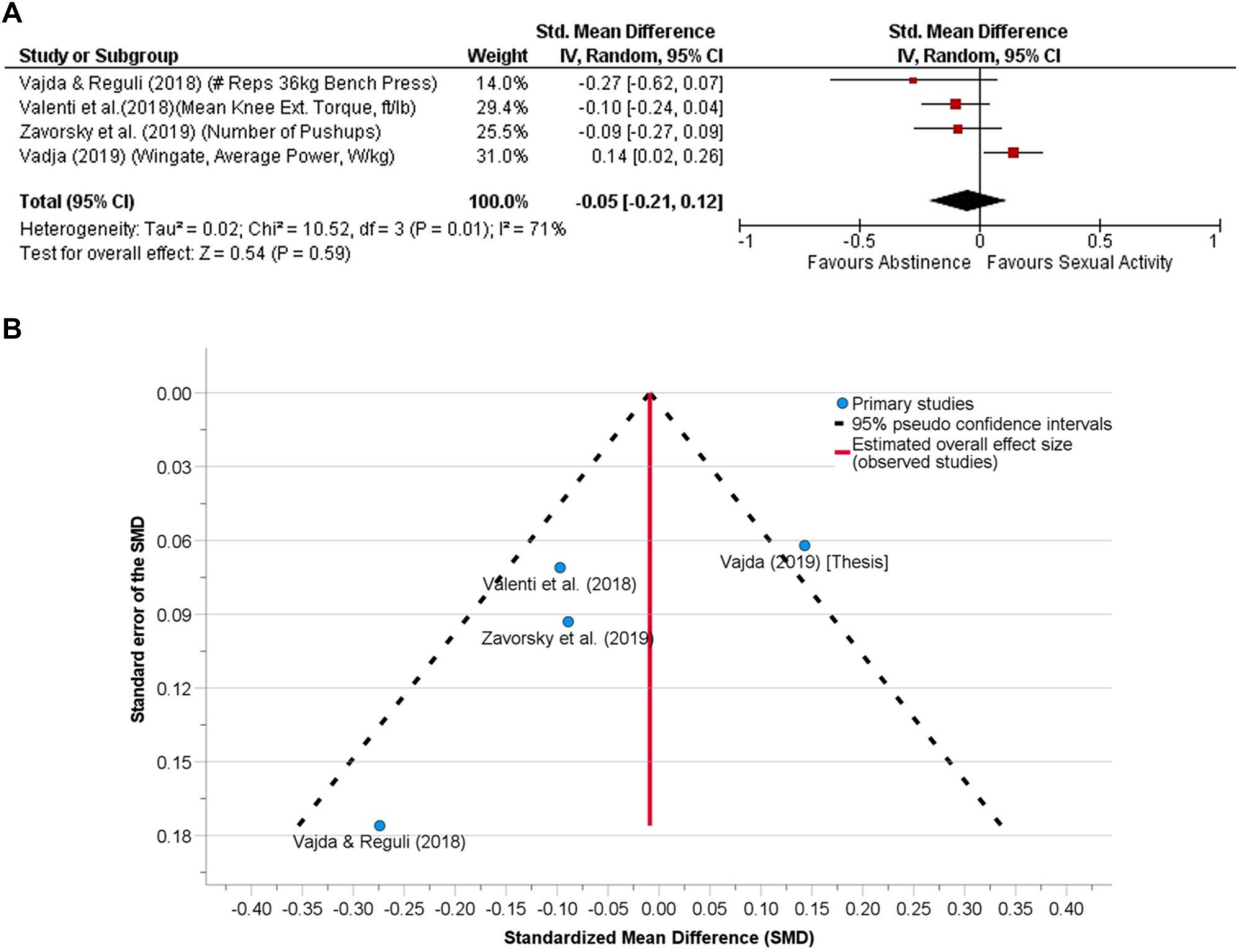
**(A)** The effect of sexual activity that occurred 7.5–24 h before assessing musculoskeletal fitness is displayed using a Forest Plot. The data demonstrate that musculoskeletal endurance is unlikely affected by prior sexual activity or abstinence. That is, both experimental and control conditions provide similar test results. A random-effects model was used. No other adjustments were made here. There was substantial heterogeneity between studies (*p* = 0.01). **(B)** Funnel plot for the detection of publication bias in (**A**). Egger’s random effects meta-regression-based test was used with the Knapp-Hartung SE adjustment. There was no funnel plot asymmetry (Intercept = 0.24, SE = 0.418, t = 1.36, *p* = 0.31, 95% CI of the coefficient = − 0.51 to 10.99); Standard error of the effect size = − 3.20, SE = 1.97, t = − 1.62, *p* = 0.25, 95% CI − 11.69 to 5.29). After adjusting for the missing studies, the SMD (SE of the SMD) was − 0.01 (0.09) [95% CI − 0.25 to 0.23, *p* = 0.92], demonstrating that neither abstinence nor sexual activity prior to exercise affects musculoskeletal fitness assessment.

**Figure 3:**
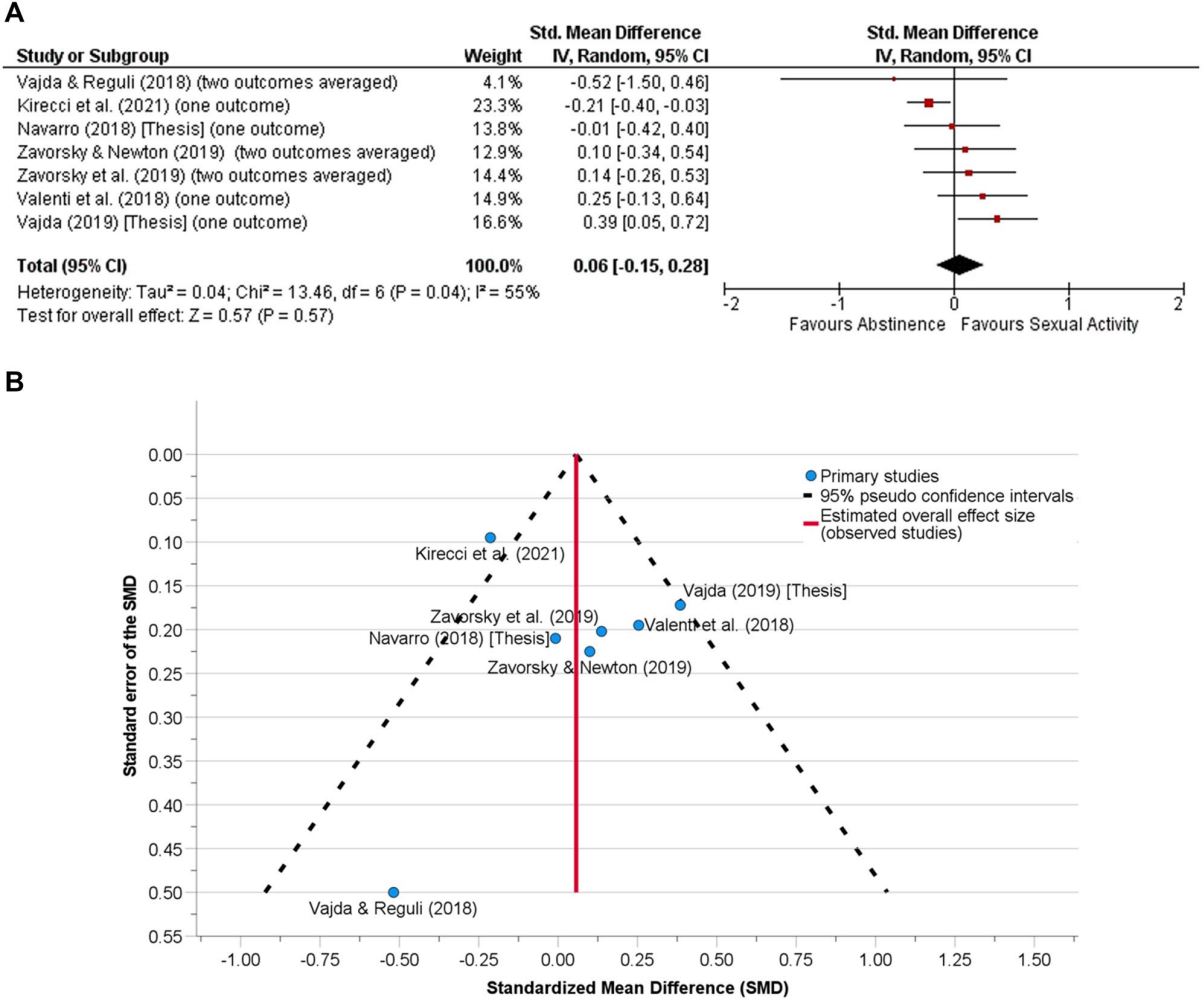
**(A)** The effect of sexual activity that occurred 30 min to 24 h before assessing muscular strength or power is displayed using a Forest Plot. There were two outcomes each for Zavorsky and Newton^44^ (n = 10), Zavorsky et al*.*^45^ (n = 7), and Vajda and Rejuli^51^ (n = 2); thus, the standardized mean difference (SMD) and standard error of the SMD was averaged for each of these studies. The data demonstrate that muscular strength or power is unlikely affected by prior sexual activity or abstinence (*p* = 0.57). That is, both experimental and control conditions provide similar test results. A random-effects model was used. No other adjustments were made here. There was substantial heterogeneity between studies (*p* = 0.04). (**B**) Funnel plot for the detection of publication bias in (**A**). Egger’s random effects meta-regression-based test was used with the Knapp-Hartung SE adjustment. There was no funnel plot asymmetry (Intercept = 0.05, SE = 0.27, t = 0.20, *p* = 0.85, 95% CI of the coefficient = − 0.65 to 0.75); Standard error of the effect size = 0.02, SE = 1.38, t = 0.02, *p* = 0.99, 95% CI − 3.53 to 3.58). After adjusting for the missing studies, the SMD (SE of the SMD) was the same as (**A**), demonstrating that muscular strength or power is unlikely affected by prior sexual activity.

**Figure 4:**
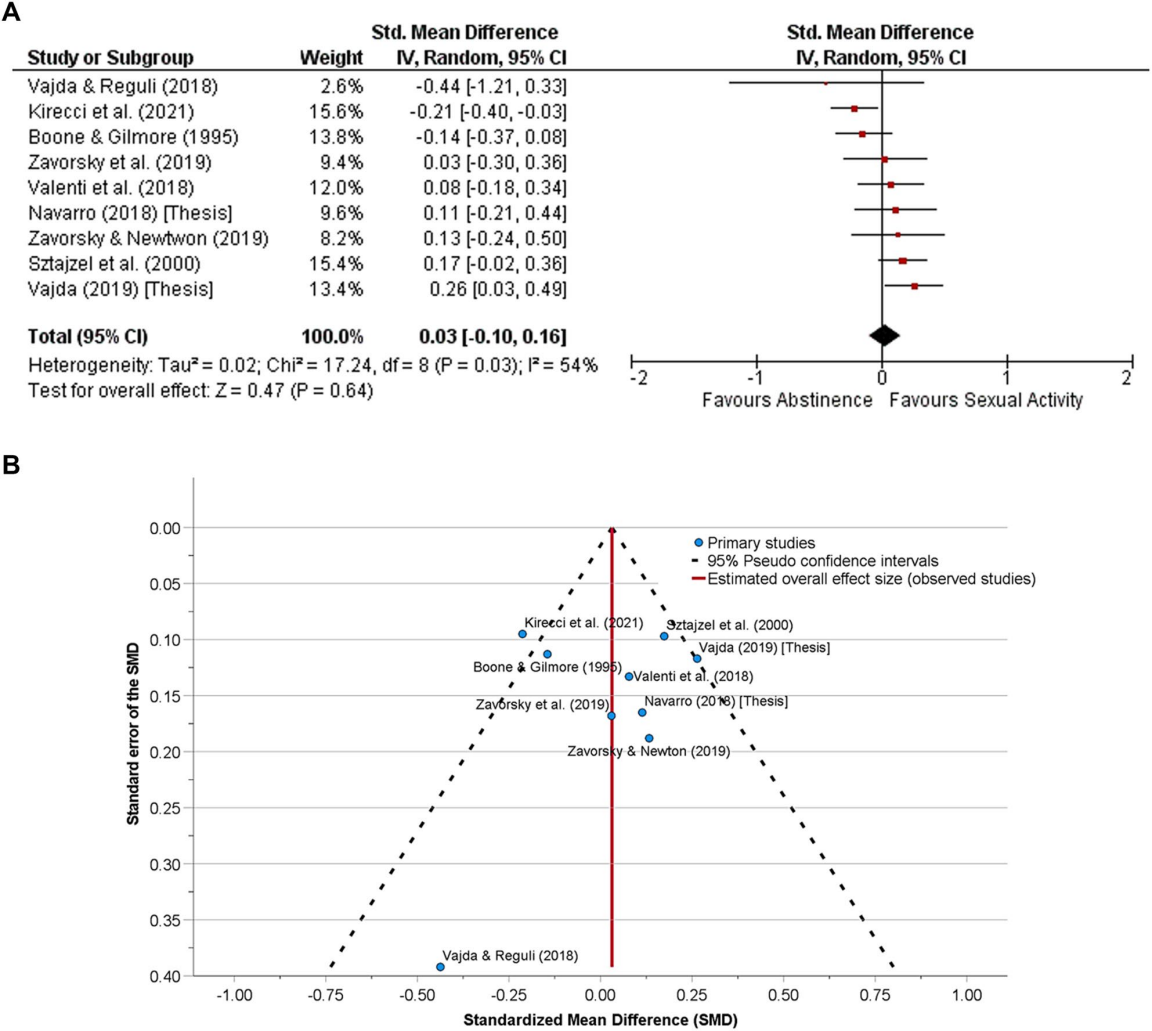
**(A)** The effect of sexual activity that occurred 30 min to 24 h before any type of physical fitness assessment (aerobic, musculoskeletal endurance, and strength/power) are summarized in this Forest Plot. There were three outcomes for Zavorsky and Newton^44^ (n = 10), four outcomes for Zavorsky et al*.*^45^ (n = 7), three outcomes for Vajda and Rejuli^51^ (n = 2), two outcomes for Valenti et al.^52^ (n = 12), and two outcomes for Navarro^43^ (n = 10); thus, the average standardized mean difference (SMD) and standard error of the SMD was used for each study with multiple outcomes in this plot. The remaining studies had one outcome. The data demonstrate that any physical performance measure is unlikely affected by either prior sexual activity or abstinence (*p* = 0.64). That is, both experimental and control conditions provide similar test results. No other adjustments were made here. There was substantial heterogeneity between studies (*p* = 0.03). (**B)** Funnel plot for the detection of publication bias in (**A**). Egger’s random effects meta-regression-based test was used with the Knapp-Hartung SE adjustment. There was no funnel plot asymmetry (Intercept = 0.10, SE = 0.19, t = 0.55, *p* = 0.60, 95% CI of the coefficient = − 0.34 to 0.55); Standard error of the effect size = − 0.55, SE = 1.34, t = − 0.41, *p* = 0.70, 95% CI − 3.71 to 2.62). After adjusting for the missing studies, the SMD (SE of the SMD) was similar to (**A**), demonstrating that muscular strength or power is unlikely affected by prior sexual activity.

Sexual activity prior to an assessment of aerobic capacity/aerobic fitness (Fig. 1A, n = 51 subjects), musculoskeletal endurance (Fig. 2A, n = 37 subjects) or muscular strength/power (Fig. 3A, n = 101 subjects) showed no difference compared to the abstinence condition. When all the data were pooled together, sexual activity 30 min to 24 h before any physical performance assessment was no different from abstinence (Fig. 4A, n = 133).

Funnel plot were presented to assess publication bias (Figs. 1B, 2B, 3B, 4B). Egger's test revealed no publication bias across studies.

Galbraith plots (Figures S2–S5) demonstrate that there was no overall difference between studies. Most points scattered homoscedastically, with unit standard deviation about the line through the origin.

**Figure 5:**
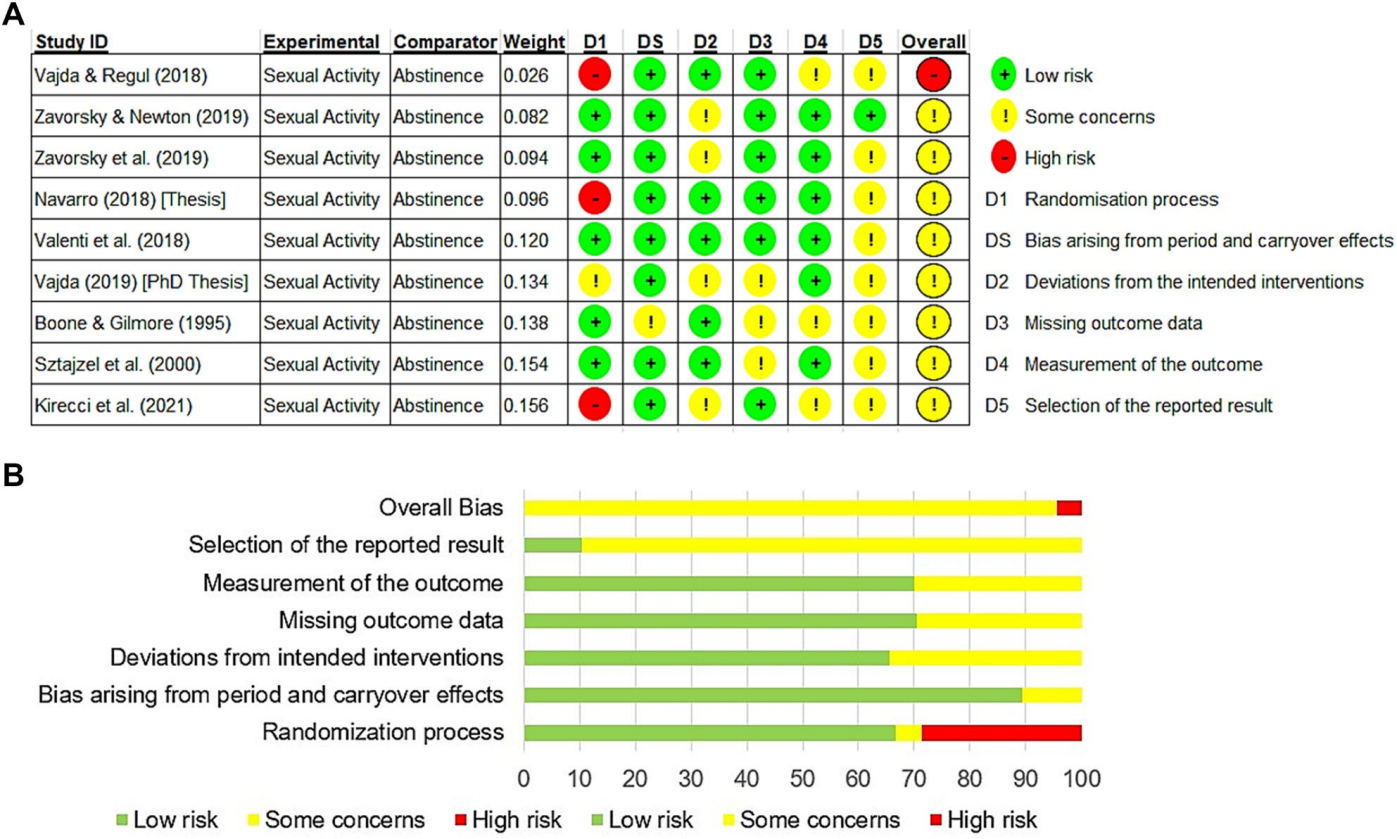
**(A)** Cochrane-style risk of bias assessment using traffic light plots displays the overall judgments by study-by-study. **(B)** Risk of bias graph as a percentage (per protocol). The six domains of bias, including overall bias, are presented.

Assessing risk of bias was determined by a revised version of the Cochrane risk of bias (RoB 2)^33^, of which an excel software tool was used for its implementation^34^. The approach used to assess bias was based on the "per protocol' effect. Individual studies were evaluated in six different domains of bias (Fig. 5A). There were some concerns for overall bias in the six domains (Fig. 5B), likely because it is impossible to blind subjects to experimental and control groups in studies of this nature. There was an overall high risk of bias in the randomization process in three studies^43,50,51^.

## Discussion

This meta-analysis sought to determine whether sexual activity before an athletic competition would be disadvantageous, beneficial, or have no impact on athletic performance. Although no study has examined the effects of sexual activity in actual competition, nine studies (including two theses) were identified in this analysis that examined the effects of sexual activity on various physical fitness tests. These results demonstrate that the outcome was similar irrespective of the type of physical fitness test used to assess performance. Physical tests that assessed aerobic capacity, musculoskeletal endurance, or muscular strength/power were not affected by prior sexual activity 30 min to 24 h before assessment. This meta-analysis is the first to investigate treatment effects between studies.

A funnel plot was created to include all assessments of physical performance (Fig. 4B). Clarivate's Web of Science™ and Google scholar was used to search the literature, but only nine studies were found that had data. Funnel plots are ill-advised with fewer than ten studies due to a high likelihood of a low statistical power distinguishing chance from real asymmetry^53^. However, the shape of our funnel plots was adjusted for missing studies using the Trim and Fill method^38^, and we believe that this was adequate to illustrate no publication bias.

There are inherent limitations to conducting a study to examine the effect of prior sexual activity on athletic performance. First, it is impossible to blind the subject to the treatment condition. Thus, the subjects' prior beliefs about the study outcome could introduce some bias. There is no way around this lack of blinding, which plays a role in the overall risk of bias in this meta-analysis (Fig. 5A, B). Another limitation is the sample size for each study is small, with one study having a sample size of two subjects^51^, while most of the other studies had between eight and 16 subjects each (Table S2). The lack of a larger sample size per study may be due to the nature of the research question, as there is difficulty in recruiting willing participants. The crossover design of many of these studies also allowed for better assessment with a smaller number of subjects. However, when pooling all the data in the literature, 134 subjects were evaluated, which increases the confidence of the findings. A third limitation is that young adult subjects were used only, and it may be hard to generalize across older adult groups. As a related issue, nearly all subjects were men, and this leaves a significant gap in understanding the effects of prior sexual activity in athletic performance in females. A fifth limitation is that no study examined the effects of prior sexual activity on actual athletic performance in competition. The use of physical performance tests as a proxy to athletic competition may not be equivalent, and a true measurement of athletic performance is needed (i.e., swimming times obtained in competition, running times obtained at track meets, etc.). There is one study published in an obscure online journal reporting no correlation between the number of sexual events engaged in 48 h before running a marathon and running performance^14^; however, that study did not specify the marathon times or the number of sexual events within the previous 48 h before the marathon.

Sexual activity is a complex interplay of physical, emotional, psychological, and factors that may be difficult to understand fully. In our pooled assessment, physical performance was not affected by sexual activity 30 min to 24 h before testing. The "inverted U" theory of arousal shows that athletic performance improves as arousal levels increase, but up to a point. Any increase in arousal above a threshold will worsen performance^54^. Therefore, sex before competition could benefit responders and be disadvantageous to nonresponders^18^. Thus, assessing athletic performance "once" in each condition could potentially show no effect. It is also possible that other factors not yet understood could be contributing to these findings.

### Opportunities based on identified gaps in the literature

Based on these findings, future studies on this topic would benefit from the following:

- Women must be studied.
- Older subjects should be investigated.
- Actual measures of athletic performance are needed. For example, running times from track competitions, swimming times from swim meets, etc.
- Larger sample sizes per trial are needed.
- Repeated assessments in each condition are needed. Studies that involve a repeated-measures, crossover design, where athletes are tested several times following abstinence and several times following sex, would be welcomed. That way, one would separate responders from non-responders^18^.
- De-emphasize the outcome being studied compared to other variables to decrease the risk of conscious and unconscious bias of the participants. This would require careful consideration and IRB approval. According to the Tri-Council Policy Statement of Ethical Conduct for Research Involving Humans^55^, this approach can be used in the research design if the research involves minimal risk to the participants and the alteration to consent requirements is unlikely to affect the welfare of the participants adversely. Furthermore, the researcher would be required to disclose the true nature of the study after study completion^55^.
- Double-blinded studies would be ideal, though it is challenging if not impossible for participants to be blinded to their sexual activity. At a minimum, researchers and individuals conducting the testing (i.e., data collectors) must be blinded to the experimental and control conditions until after the statistical analysis, and this must be explicitly stated.
- Attempt to control for potential confounders. For example, is it the act of sexual intercourse or masturbation that does or does not impact performance, or, rather is it the orgasm that occurs and subsequent hormonal alterations that can or does not impact performance? One example is adding a Yoga group or other type of activity with equivalent energy expenditure to control for the energetic effects of exercise and/or lack of orgasm^45^.
- Time between sexual activity and competition should be standardized. The meta-analysis shows large variability in the lapse in time between sexual activity and assessing a physical performance measure. Studies should further examine whether this lapse in time between sex and exercise can affect outcomes.

## Conclusion

Performance in several physical fitness measures was unaltered in young men after sexual activity that occurred in the previous 30 min to 24 h before the assessment. There was no publication bias present; however, there was substantial heterogeneity between studies. There was some concern about the overall risk of bias, partly due to the subjects knowing when they are exposed to the experimental and control arms. Future work in this area is needed to mitigate some of these limitations.

## Supplementary Information

Supplementary Information.

## Data Availability

The aggregate data is available in the online supplement.
