# Supplementary material for: The influence of sexual activity on athletic performance: a systematic review and meta-analyses
Source: Sci Rep. 2022 Sep 16;12:15609. doi: 10.1038/s41598-022-19882-2 (PMC9481637; doi:10.1038/s41598-022-19882-2)
Supplement: Supplementary file 1 — Supplementary Information. [file 41598_2022_19882_MOESM1_ESM.pdf]

ONLINE SUPPLEMENTARY MATERIAL

The influence of sexual activity on athletic performance: A systematic review and meta-analyses

[1]Gerald S. Zavorsky, [2]Rebecca A. Brooks

[1]Department of Physiology and Membrane Biology, University of California, Davis, United States

[2]Division of Gynecologic Oncology, University of California, Davis, United States

Gerald S. Zavorsky ORCID ID: 0000-0002-4473-1601

Rebecca A. Brooks ORCID ID: 0000-0001-9185-8501

This supplement has been peer-reviewed.

Correspondence:
Gerald S. Zavorsky, Ph.D., FACSM
Department of Physiology and Membrane Biology
University of California, Davis
e-mail: gszavorsky@ucdavis.edu
gerryzavorsky@gmail.com

Table S1. The mathematical formulas used for cross-over trials[1].

Mean difference<br>$\mathrm{MD} = M_E - M_C$	SE of the mean difference<br>$\mathrm{SE(MD)} = \frac{\mathrm{SD_{diff}}}{\sqrt{N}}$
Estimated SD of the differences<br>$\mathrm{SD_{diff}} = \sqrt{\mathrm{SD}_E^2 + \mathrm{SD}_C^2 - (2 \times \mathrm{Corr} \times \mathrm{SD}_E \times \mathrm{SD}_C)}$	Standardized mean difference (SMD)<br>$\mathrm{SMD} = \frac{\mathrm{MD}}{\mathrm{SD_{pooled}}}$
Pooled SD<br>$\mathrm{SD_{pooled}} = \sqrt{\frac{\mathrm{SD}_E^2 + \mathrm{SD}_C^2}{2}}$	Standard error (SE) of the standardized mean difference (SMD)<br>$\mathrm{SE(SMD)} = \sqrt{\frac{1}{N} + \frac{\mathrm{SMD}^2}{2N}} \times \sqrt{2(1 - \mathrm{Corr})}$
Standardized mean difference (SMD)<br>$\mathrm{SMD} = \frac{\mathrm{MD}}{\mathrm{SE(MD)} \times \sqrt{\frac{N}{2(1 - \mathrm{Corr})}}}$	Imputed correlation coefficient (only used when raw data is not provided to calculate the true correlation coefficient)<br>$\mathrm{Corr} = \frac{\mathrm{SD}_E^2 + \mathrm{SD}_C^2 - \mathrm{SD}_{\mathrm{diff}}^2}{2 \times \mathrm{SD}_E \times \mathrm{SD}_C}$

M_E = mean experimental; M_C = Mean control ; SD_E = Standard deviation experimental; SD_C = Standard deviation control ; Corr = correlation.

Table S2. Studies identified that examined if prior sexual activity affected physical performance.

Study	Number of healthy, physically active male subjects per study	Age (yrs)	Time between sexual activity & performance testing	Wash-out period
**Kirecci *et al.* (2021)**[2]	50 males	29 (1)	24 hrs	7 days
**Zavorsky *et al.* (2019)**[3]	6 males + 1 female	28 (5)	7.5 hrs	5-7 days
**Zavorsky & Newton (2019)**[4]	10 males	28 (7)	8 hrs	5 days
**Vajda (2019) [PhD Thesis]**[5]	16 males	23 (2)	24 hrs	1 day
**Valenti *et al.* (2018)**[6]	12 males	26 (4)	12 hrs	3-7 days
**Navarro (2018)* [Physiotherapy Thesis]**[7]	10 males	22 (2)	30 minutes	7 days
**Vajda & Reguli (2018)**[8]	2 males	26 (0)	12 hrs	1 day
**Sztajzel *et al.* (2000)**[9]	15 males	29 (6)	2.5 hrs	2 days
**Boone & Gilmore (1995)**[10]	11 males	26 (2)	12 hrs	5-7 days

Note: all but one of the 133 subjects were healthy, physically active males. Parentheses indicate the standard deviation *The Navarro study had subjects masturbate before exercise, while the participants from the other studies had sexual intercourse with a partner. Also, the two published papers by Zavorsky were based on master theses[11 12]. One study was orally presented at a congress in 1989[13], but it was published 11 years later [9].

Table S3. Displayed physical performance measurements from each study in both control (abstinence) and experimental (sexual activity) conditions. There were nine studies in this meta-analysis[2-10], and some studies used several different performance measures.

The type of physical performance test used in each study	Mean Abstinence	SD Abstinence	Mean Sexual Activity	SD Sexual Activity	Pooled SD	Mean Difference (Sex Activity – Abstinence)	SD of the mean difference	SE of the mean difference	Imputed or measured correlation coefficients	SMD (Hedges g)	SE of the SMD
Zavorsky *et al.* (2019) (number of pushups to failure)	33.4	15.3	32	16.0	15.7	-1.4	3.66	1.4	0.97	-0.089	0.093
Zavorsky *et al.* (2019) (handgrip strength, psi)	19.5	3.3	20.5	3.3	3.3	1.0	1.2	0.5	0.94	0.30	0.134
Zavorsky et al. (2019) (wattage that elicits a heart rate of 170 beats/min)	111	31	109	32	31.50	-2.00	15.90	6.49	0.91	-0.06	0.173
Zavorsky *et al.* (2019) (standing vertical jump height, cm)	44.9	14.2	44.5	12.5	13.4	-0.4	8.6	3.5	0.78	-0.03	0.271
Zavorsky & Newton (2019) Margaria-Kalamen power test, W)	216	28	218	26	27.0	2.0	9.0	2.8	0.95	0.07	0.105
Zavorsky & Newton (2019) (VO_{2max}, mL/kg/min)	54.6	2.5	55.5	5.9	4.53	0.90	3.70	1.23	0.94	-0.20	0.114
Zavorsky & Newton (2019) Handgrip strength, kg)	50	6.1	50.7	4.9	5.5	0.7	6.1	1.9	0.41	0.13	0.345
Vajda (2019) [PhD Thesis]. (Average Wingate power, w/kg)	8.9	0.7	9	0.7	0.7	0.1	0.17	0.04	0.97	0.143	0.062
Vajda (2019) [PhD Thesis](Max Wingate power, w/kg)	12.5	1.3	13	1.3	1.3	0.5	0.9	0.2	0.78	0.38	0.172

Table S3 continued

The type of physical performance test used in each study	Mean Abstinence	SD Abstinence	Mean Sexual Activity	SD Sexual Activity	Pooled SD	Mean Difference (Sex Activity – Abstinence)	SD of the mean difference	SE of the mean difference	Imputed or measured correlation coefficients	SMD (Hedges g)	SE of the SMD
Valenti *et al.* (2018) (mean knee extension torque, ft/lb) (set #5)	153	32	150	30	31.0	-3.0	7.8	2.3	0.97	-0.097	0.071
Valenti *et al.* (2018) (Peak knee extension torque, ft/lb) (set #5)	190.2	28.7	198.9	39.1	34.3	8.7	24.5	7.1	0.78	0.25	0.195
Kirecci et al. (2021) (mean weight pushed for 5 sets of 5 reps, with 1 min rest between sets)	109.4	11.4	107	11.1	11.3	-2.4	7.5	1.1	0.78	-0.21	0.095
Sztajzel *et al.* (2000) (VO_{2max}, L/min)	4.55	0.55	4.65	0.6	0.58	0.10	0.23	0.06	0.93	0.17	0.099
Boone & Gilmore (1995) (VO_{2max}, L/min)	3.53	0.7	3.43	0.68	0.69	-0.10	0.26	0.08	0.93	0.14	0.116
Vajda & Reguli (2018) (Number of reps bench press to failure using 36 kg weight)	87	48.1	75.5	34.6	41.9	-11.5	13.4	9.5	0.97	-0.274	0.176
Vajda & Reguli (2018) (Handgrip strength, N)	630.5	82.7	588	111.7	98.3	-42.5	29.0	20.5	0.78	-0.43	0.490
Vajda & Reguli (2018) (Wingate, peak power, W/kg)	12.7	1.3	12	1	1.2	-0.7	2.3	1.6	0.78	-0.60	0.510
Navarro (2018) [Physiotherapy Thesis] (peak wattage from VO_{2max} test)	318.5	30.7	325.5	28.8	29.8	7.0	11.3	3.6	0.93	0.24	0.12
Navarro (2018) [Physiotherapy Thesis] (Handgrip strength, kg)	54.7	12.8	54.6	12.7	12.8	-0.1	8.5	2.67	0.78	-0.01	0.4

SD = standard deviation SMD = standardized mean difference; SE = standard error; the control treatment was abstinence; the experimental treatment was sexual activity.

Figure S1. PRISMA Diagram[14]. The flow of information through the different phases of a systematic review. It maps out the number of records identified, included, and excluded, and the reasons for exclusion.

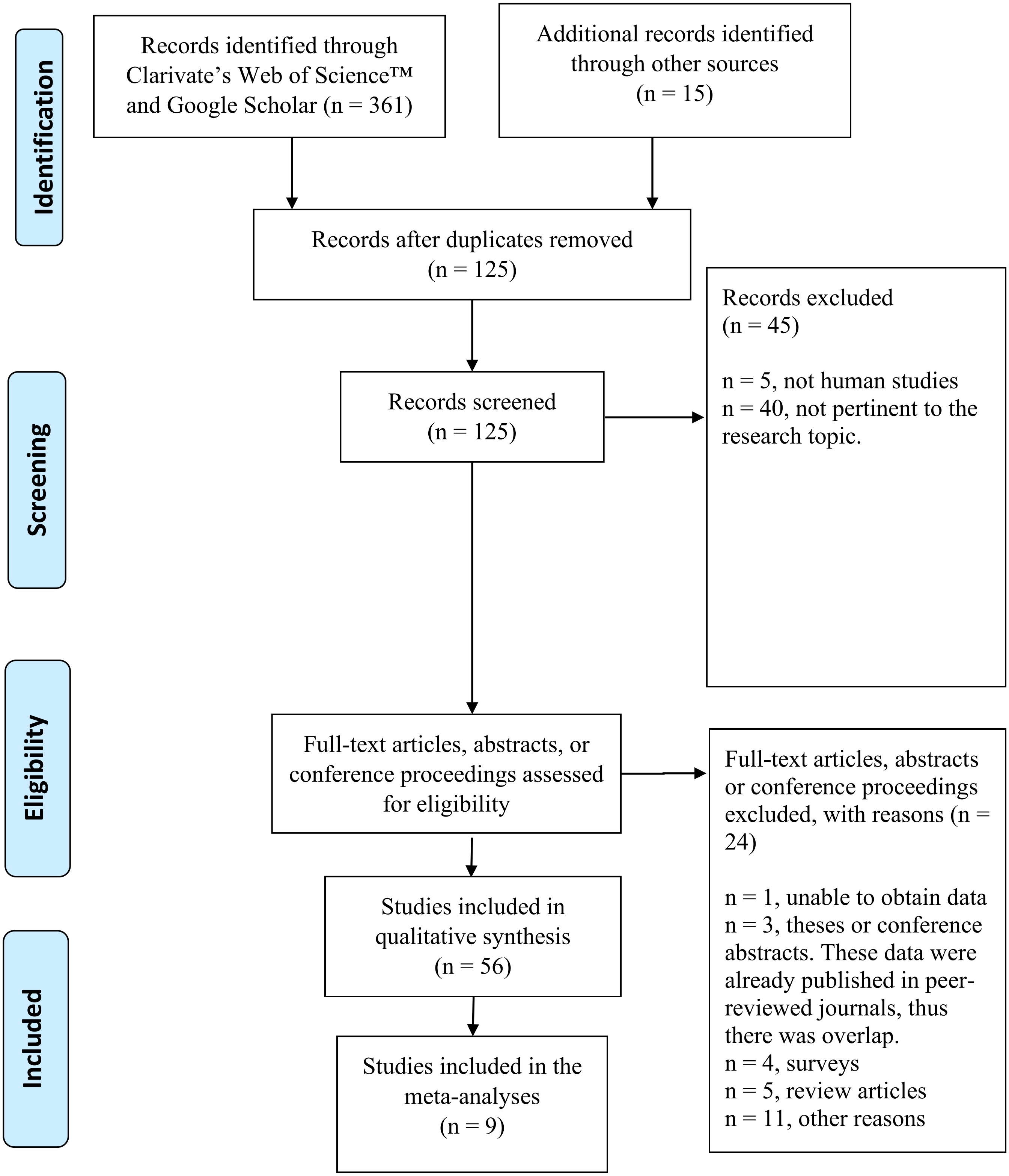

Figure S2. The effect of sexual activity that occurred 30 minutes to 12 hours before an aerobic fitness assessment is displayed using a Galbraith plot[15]. With aerobic fitness performance measures converted to z-scores (y-axis) plotted against the inverse of the standard error (x-axis), it can be seen that there is no overall difference between studies. The points scatter homoscedastically, with unit standard deviation about the line through the origin.

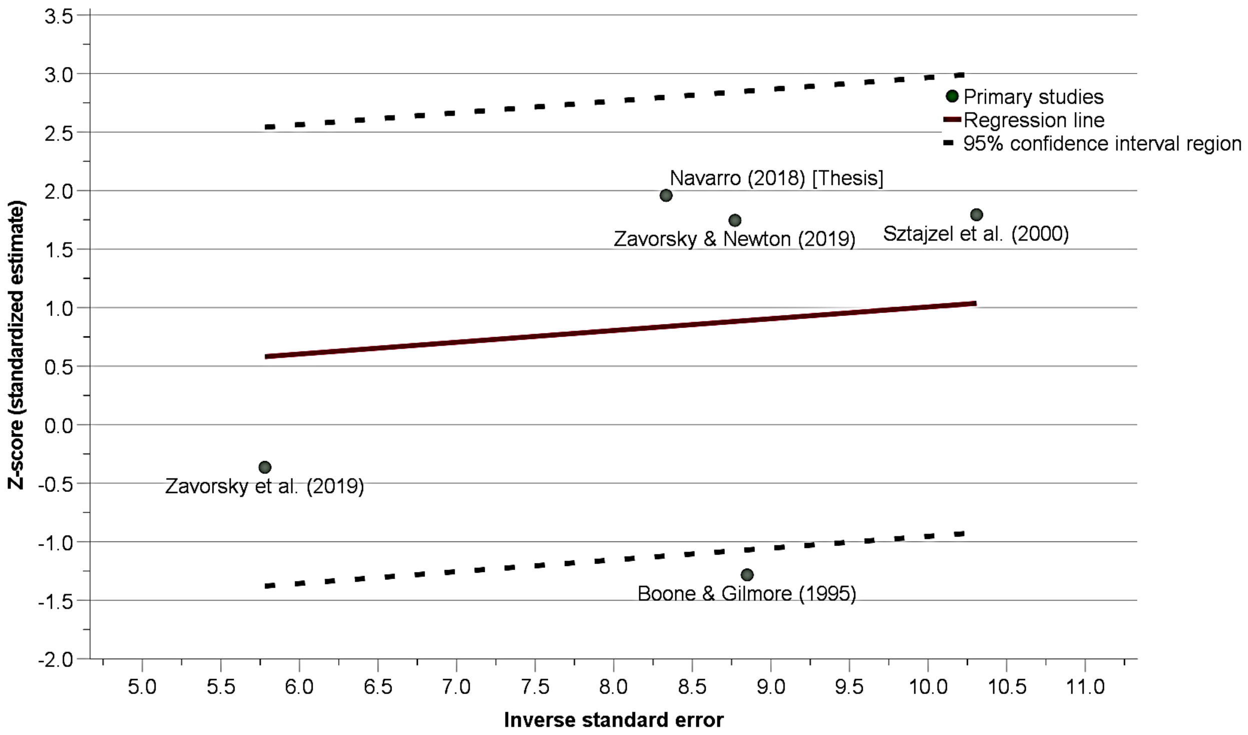

Figure S3. The effect of sexual activity that occurred 7.5 to 24 hours before assessing musculoskeletal fitness is displayed using a Galbraith plot[15]. With musculoskeletal fitness performance measures converted to z-scores (y-axis) plotted against the inverse of the standard error (x-axis), it can be seen that there is no overall difference between studies. The points scatter homoscedastically, with unit standard deviation about the line through the origin.

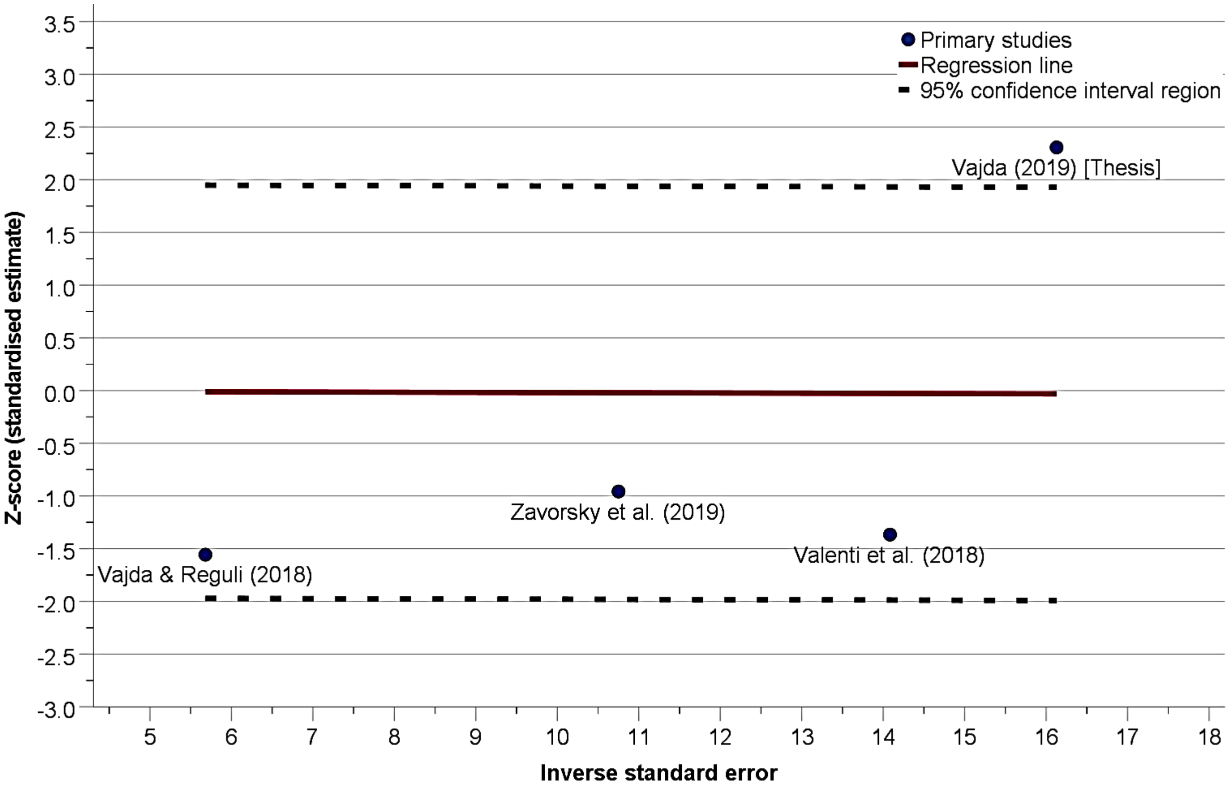

Figure S4. The effect of sexual activity that occurred 30 minutes to 24 hours before assessing muscular strength or power is displayed using a Galbraith plot[15]. With muscular strength / power performance measures converted to z-scores (y-axis) plotted against the inverse of the standard error (x-axis), it can be seen that there is no overall difference between studies. The points scatter homoscedastically, with unit standard deviation about the line through the origin.

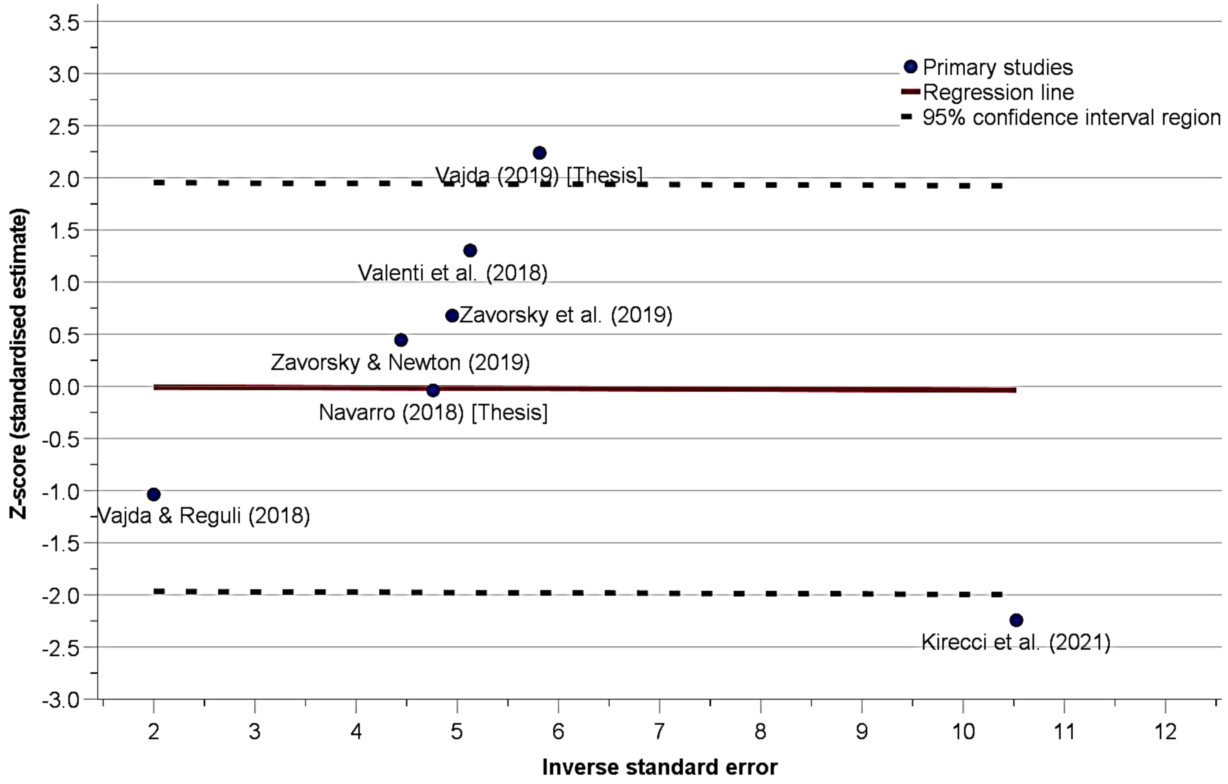

Figure S5. The effect of sexual activity that occurred 30 minutes to 24 hours before assessing any type of physical fitness assessment is displayed using a Galbraith plot[15]. That is, the results of aerobic, musculoskeletal endurance, and strength/power outcomes are summarized here. With performance measures converted to z-scores (y-axis) plotted against the inverse of the standard error (x-axis), it can be seen that there is no overall difference between studies. The points scatter homoscedastically, with unit standard deviation about the line through the origin.

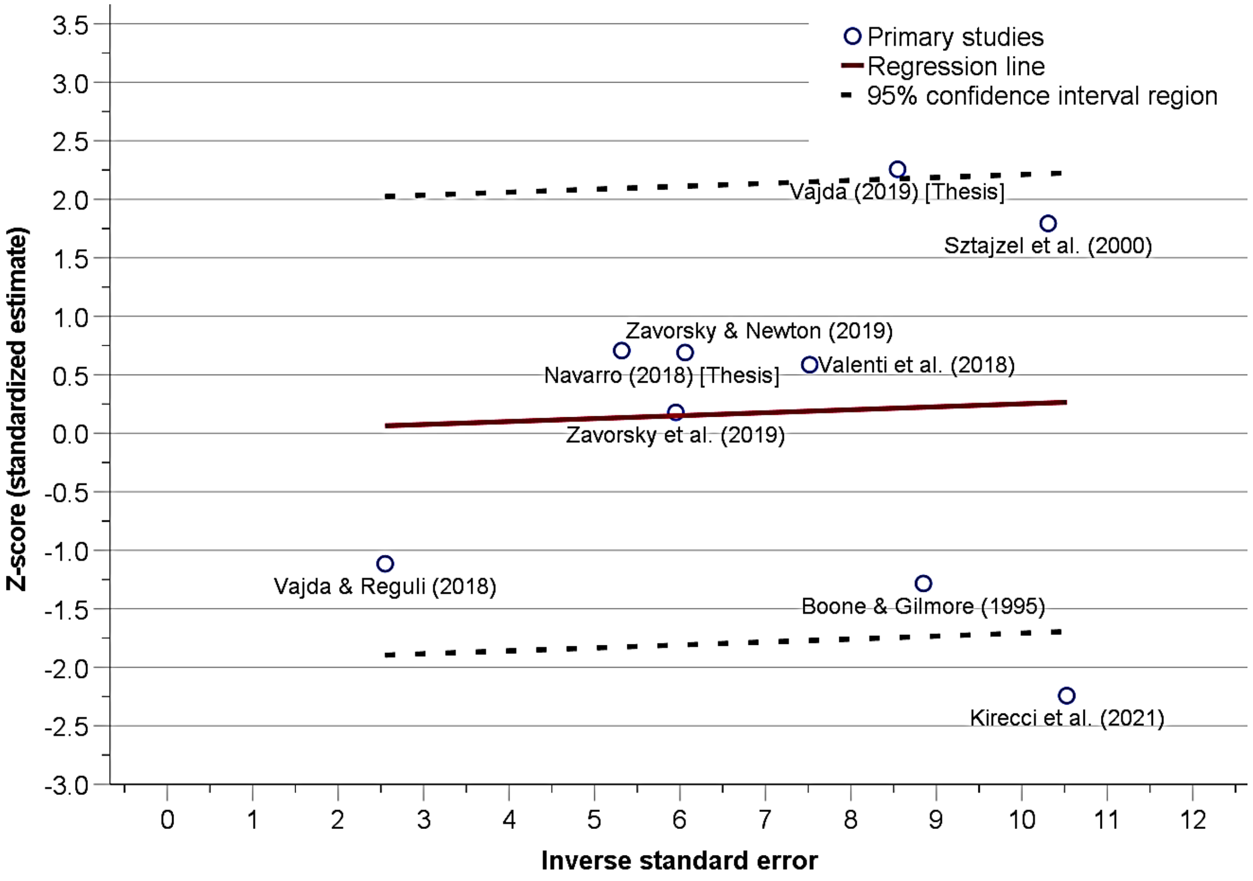

References

1. Higgins JPT, Eldridge SM, Li T. Including variants on randomized trials. In: Higgins JPT, Thomas J, Chandler J, et al., eds. Cochrane Handbook for Systematic Reviews of Interventions (version 62) The Cochrane Collaboration 2021:26 pages.

2. Kirecci SL, Albayrak AT, Yavuzsan AH, et al. Sexual intercourse before exercise has a detrimental effect on lower extremity muscle strength in men. *Postgrad Med J* 2021 doi: 10.1136/postgradmedj-2020-139033 [published Online First: 2021/04/03]

3. Zavorsky GS, Vouyoukas E, Pfaus JG. Sexual activity the night before exercise does not affect various measures of physical exercise performance. *Sex Med* 2019;7(2):235-40. doi: 10.1016/j.esxm.2018.12.002

4. Zavorsky GS, Newton WL. Effects of sexual activity on several measures of physical performance in young adult males. *J Sports Med Phys Fitness* 2019;59(7):1102-09. doi: 10.23736/S0022-4707.18.09070-9

5. Vajda P. Effect of sexual abstinence and activity on performance in leisure sports [PhD Thesis]. Masaryk University, Brno, Czech Republic, 2019.

6. Valenti LM, Suchil C, Beltran G, et al. Effect of Sexual Intercourse on Lower Extremity Muscle Force in Strength-Trained Men. *J Sex Med* 2018;15(6):888-93. doi: 10.1016/j.jsxm.2018.04.636

7. Navarro JC. Sexual activity, sport, and quality of life, related to health [Physiotherapy Thesis]. University of Valladolid, Valladolid, Spain, 2018.

8. Vajda P, Reguli Z. Effect of sexual abstinence on physical performance in combat sports: a pilot study. *Journal of Physical Education and Sport* 2018;18(4):2303-07. doi: 10.7752/jpes.2018.04347

9. Sztajzel J, Periat M, Marti V, et al. Effect of sexual activity on cycle ergometer stress test parameters, on plasmatic testosterone levels and on concentration capacity. A study in high-level male athletes performed in the laboratory. *Journal of Sports Medicine and Physical Fitness* 2000;40(3):233-9.

10. Boone T, Gilmore S. Effects of sexual intercourse on maximal aerobic power, oxygen pulse, and double product in male sedentary subjects. *Journal of Sports Medicine and Physical Fitness* 1995;35(3):214-7.

11. Newton WL. Effects of sexual activity on performance of physical capacity tests in young, fit males. [Masters Thesis]. Colorado State Univesity, Fort Collins, United States, 1987.

12. Vouyoukas E. The influence of sexual activity on athletic performance. [Masters Thesis] Concordia University, Montreal, Canada, 2011.

13. Sztajzel J, Periat M, Krall P, et al. Does sexual activity have an effect on athletic performance? [abstract]. 11th Congress of the European Society of Cardiology, 10–14 September 1989, Nice, France: day 1, monday, 11 September 1989. *Eur Heart J* 1989;10:85.

14. Moher D, Liberati A, Tetzlaff J, et al. Preferred reporting items for systematic reviews and meta-analyses: the PRISMA statement. *PLoS Med* 2009;6(7):e1000097. doi: 10.1371/journal.pmed.1000097

15. Galbraith RF, Galbraith JI. On the graphical presentation of a collection of means. *J Roy Stat Soc a Sta* 1996;159:611-13.
